# Effects of a Xenogeneic Bone Graft Combined with Platelet-Rich Fibrin Obtained Using Two Distinct Centrifugation Protocols on Critical-Size Rat Calvarial Defects: A Microtomographic, Histomorphometric, and Confocal Laser Scanning Microscopy Analysis

**DOI:** 10.3390/jfb17090451

**Published:** 2026-09-06

**Authors:** Ulli da Costa Cunha Martins, Débora de Souza Ferreira Sávio, Roberta Okamoto, Carlos Fernando Mourão, Richard J. Miron, Sérgio Luis Scombatti de Souza, Flávia Aparecida Chaves Furlaneto, Michel Reis Messora

**Affiliations:** 1 Department of Oral and Maxillofacial Surgery and Periodontology (DCTBMF), School of Dentistry of Ribeirão Preto, University of São Paulo (USP), Ribeirão Preto 14040-904, SP, Brazil; ullicostamartins@gmail.com (U.d.C.C.M.); deborasouzaf@hotmail.com (D.d.S.F.S.); carlos.mourao@tufts.edu (C.F.M.); scombatti@forp.usp.br (S.L.S.d.S.); flafurlaneto@usp.br (F.A.C.F.); 2 Department of Basic Sciences (DCB), School of Dentistry of Araçatuba, São Paulo State University (UNESP), Araçatuba 16015-050, SP, Brazil; roberta.okamoto@unesp.br; 3 Department of Basic and Clinical Translational Sciences, Tufts University School of Dental Medicine, Boston, MA 02111, USA; 4 Department of Periodontology, School of Dental Medicine, University of Bern, 3010 Bern, Switzerland; rick@themironlab.com

**Keywords:** bone regeneration, platelet-rich fibrin, bone graft, horizontal centrifugation, micro-computed tomography

## Abstract

This study aimed to evaluate bone regeneration in critical-size rat calvarial defects treated with a xenogeneic bone graft alone or combined with autologous platelet concentrates prepared using two distinct centrifugation protocols, one based on horizontal centrifugation to produce horizontal platelet-rich fibrin (H-PRF) and the other based on vertical fixed-angle centrifugation to produce leukocyte–platelet-rich fibrin (L-PRF). Calvarial defects were created in 24 rats and allocated to four groups: blood clot (C), xenograft (XEN), xenograft plus L-PRF (XEN+L-PRF), and xenograft plus H-PRF (XEN+H-PRF) (*n* = 6/group). Calcein and alizarin were administered at 14 and 30 days, respectively. After 35 days, specimens were analyzed by micro-computed tomography, confocal laser scanning microscopy, histomorphometry, and histopathology. Data were analyzed using ANOVA and Tukey’s post hoc test (*p* < 0.05). All treated groups showed higher bone volume and lower trabecular separation than group C. The XEN+L-PRF group showed significantly higher bone volume than the XEN group. The XEN+H-PRF group showed higher bone volume, connectivity density, alizarin labeling, mineral apposition rate, and newly formed bone area, as well as lower trabecular separation, than the other groups (*p* < 0.05). Under the experimental conditions evaluated, both PRF protocols improved bone regeneration when combined with a xenogeneic bone graft, whereas the H-PRF protocol was associated with superior structural and histomorphometric bone regeneration outcomes and with more favorable spatiotemporal mineralization kinetics.

## 1. Introduction

The development of biomaterials capable of enhancing bone regeneration remains a major focus of regenerative dentistry because of the limited healing potential of critical bone defects [1]. Among these biomaterials, autologous platelet concentrates have been widely investigated as adjuncts to bone grafting procedures owing to their ability to stabilize graft particles while providing a sustained source of growth factors and immune cells. Platelet-rich fibrin (PRF), a second-generation platelet concentrate, has demonstrated favorable effects on bone and soft tissue regeneration, including angiogenic, anti-inflammatory, and osteogenic properties, supporting its clinical use in guided bone regeneration procedures [2,3,4]. Nevertheless, strategies to further optimize the biological performance of PRF continue to be investigated.

Recent advances have focused on the influence of centrifugation protocols on the biological characteristics of PRF. Compared with conventional fixed-angle centrifugation used to produce leukocyte–platelet-rich fibrin (L-PRF), horizontal centrifugation has been shown to yield higher cellular content, a more homogeneous distribution of platelets and leukocytes, enhanced growth factor release, and a denser fibrin architecture [5,6,7]. The three-dimensional organization of the PRF matrix and the distribution of cellular components within the fibrin architecture may also influence its biological behavior [7]. These differences are thought to result from the distinct orientation of blood components during centrifugation, which may reduce cell deformation caused by friction against the tube walls [4]. Consistent with these biological findings, a previous study demonstrated that horizontal PRF (H-PRF) resulted in superior regenerative outcomes compared with L-PRF in an experimental model of bone regeneration [8]. A clinical study also reported improved graft integration when H-PRF is combined with deproteinized bovine bone mineral in maxillary sinus augmentation procedures [9].

Despite these promising findings, evidence supporting the regenerative potential of H-PRF remains limited [6]. Most available studies have focused on the structural and biological characteristics of different PRF preparations, whereas comparatively few preclinical investigations have evaluated their effects on bone regeneration. Experimental evidence has shown that H-PRF results in greater bone formation in rat calvarial defects when compared with L-PRF and A-PRF [8]. However, its isolated use presents limitations due to the rapid degradation of the PRF matrix and limited mechanical stability, supporting its combination with bone graft materials [10,11]. To date, no preclinical studies have evaluated whether H-PRF combined with bone grafts provides more favorable regenerative outcomes than other PRF protocols.

Therefore, this study aimed to evaluate bone regeneration in critical-size rat calvarial defects treated with xenogeneic bone graft combined with autologous platelet concentrates prepared by horizontal centrifugation (H-PRF) or vertical fixed-angle centrifugation (L-PRF).

## 2. Materials and Methods

### 2.1. Animal Model

Before the start of the study, the animal management procedures and the experimental protocol were approved by the local Committee on Ethics in the Use of Animals (CEUA, protocol No. 2019.1.930.58.0, approved on 7 February 2020). Twenty-four rats (*Rattus norvegicus albinus*, Wistar; body weight 350–450 g; 14 weeks of age) obtained from the Animal Facility of the School of Dentistry of Ribeirão Preto (FORP) of the University of São Paulo (USP) were housed under controlled conditions (12 h light/dark cycle; constant temperature of 22–24 °C) and provided with standard solid chow and water ad libitum.

The sample size was determined using GraphPad StatMate 2.0 (GraphPad Software, Inc., San Diego, CA, USA), assuming an alpha level of 0.05 and a statistical power of 80%. Based on the parameters adopted for the primary outcome, bone volume fraction (BV/TV; bone volume/total volume) [12], a total of 24 animals were allocated to four experimental groups (*n* = 6 per group): control (C), xenograft (XEN), xenograft combined with L-PRF (XEN+L-PRF), and xenograft combined with H-PRF (XEN+H-PRF). Animals were randomly assigned to the experimental groups using a computer-generated sequence of random numbers generated in Microsoft Excel prepared by an investigator not involved in the subsequent experimental procedures, and housed in four cages, with six animals per cage. No predefined animal- or data-level inclusion or exclusion criteria were established, and all animals fulfilling the general eligibility requirements of the study were included in the analyses.

All investigators (operators, outcome assessors, and biostatisticians) were calibrated and blinded to group allocation. The study coordinator (M.R.M.) was aware of group allocation during the allocation and surgical procedures but did not participate in data analysis, so that blinding was maintained throughout all outcome evaluations.

### 2.2. Surgical Protocol

Anesthesia was induced by inhalation of 4% isoflurane (Instituto Biochimico Ind. Farm. Ltd.a., Itatiaia, RJ, Brazil) in an induction chamber. The animals then received morphine sulfate (8 mg/kg; Dimorf, Cristália Prod. Quím. Farm. Ltd.a., Itapira, SP, Brazil) intramuscularly, flunixin meglumine (2 mg/kg; Aplonal 1%, König, Buenos Aires, Argentina) subcutaneously, and penicillin G benzathine (24,000 IU/kg; Pentabiótico Veterinário Pequeno Porte, Fort Dodge Animal Health, Campinas, SP, Brazil) intramuscularly. The experimental surgical procedures, comprising blood collection for PRF preparation and creation of the critical-size defect (CSD), were then performed. After anesthesia and before creation of the CSD, 3.5 mL of blood was collected by cardiac puncture from the animals in the XEN+L-PRF and XEN+H-PRF groups for PRF preparation. The blood collection procedure was performed as previously described [8].

The PRF preparation was performed using two distinct protocols. L-PRF was prepared following an adjusted protocol [13] using a fixed-angle centrifuge (Intra-Spin™, Intra-Lock^®^ International, Inc., Boca Raton, FL, USA; 33° rotor angle). Blood was centrifuged at 2700 rpm for 12 min, corresponding to a maximum relative centrifugal force (RCF_max_) of approximately 700× *g*. H-PRF was prepared following an adjusted horizontal centrifugation protocol [4] using an Eppendorf 5702 centrifuge (Eppendorf, Hamburg, Germany) at an RCF_max_ of 700× *g* for 8 min. The two protocols therefore differed in centrifuge configuration and processing time, although the maximum relative centrifugal force applied was approximately equivalent. Both PRF protocols have been validated in a previous study [8].

A full-thickness flap was elevated following a semilunar skin incision with a No. 15C scalpel blade. Critical-size defects measuring 5 mm in diameter were created in the parietal region of the calvaria using a 5 mm trephine bur (Neodent^®^, Curitiba, PR, Brazil) at 1000 rpm under continuous sterile saline irrigation, and the bone segment was carefully removed with a Hollenback carver (Hollenback Sculptor No. 3SS, SSWhite, Rio de Janeiro, RJ, Brazil). The procedure was performed while preserving the integrity of the dura mater and the underlying brain tissue. The defect was considered a critical-size defect because defects of this dimension do not undergo spontaneous complete healing within the experimental period [14,15]. Two reference marks were subsequently placed 2 mm anterior and 2 mm posterior to the defect margins using amalgam (Amalgam gs-80, SDI Limited, Bayswater, VIC, Australia) to facilitate identification of the original defect boundaries during subsequent analyses.

The xenogeneic bone graft used in this study was Bio-Oss^®^ (Geistlich Pharma AG, Wolhusen, Switzerland), a deproteinized bovine bone mineral in small-granule form. In the C group, the defects were filled with the naturally formed blood clot. In the XEN group, the defects were filled with 0.02 mL of the xenograft. In the XEN+L-PRF and XEN+H-PRF groups, 0.01 mL of the xenograft was combined with 0.01 mL of the respective PRF matrix. In the PRF-treated groups, the PRF clot was compressed and the resulting membrane was divided into two portions. The portion adjacent to the red blood cell layer was fragmented into small pieces and combined with the xenogeneic graft for defect filling. The remaining portion of the PRF membrane was positioned over the defect to help contain the grafting material and minimize displacement. The graft and PRF volumes were standardized using an adapted 1 mL syringe.

Primary closure was achieved with simple interrupted sutures using 4-0 silk (Ethicon^®^, Johnson & Johnson, São José dos Campos, SP, Brazil). Postoperatively, tramadol hydrochloride (20 mg/kg) and flunixin meglumine (2 mg/kg) were administered every 12 h for 2 days. No postoperative deaths or adverse events were observed during the experimental period, and no animal or data point was excluded from the analysis. The animals were monitored daily throughout the experimental period for adverse clinical signs, including respiratory distress, lethargy, abnormal posture, inability to access food or water, and wound complications.

### 2.3. Fluorochrome Administration

Sequential fluorochrome labeling was performed to assess mineralized tissue deposition during the healing period. Calcein green (20 mg/kg) was administered intramuscularly 14 days after creation of the critical-size defects, followed by intramuscular administration of alizarin red (20 mg/kg) on day 30. The two fluorochromes allowed visualization of mineral deposition at distinct stages of the healing process [16].

### 2.4. Euthanasia

The animals were euthanized 35 days after the creation of CSD in a CO_2_ chamber at a controlled flow rate, after anesthesia with 10% ketamine (80 mg/kg) combined with 2% xylazine (10 mg/kg). Calvarial specimens, including the original surgical defect and surrounding tissues, were removed en bloc with a diamond disc and fixed in 10% neutral buffered formalin for 24 h. The specimens were then washed in running water for 24 h, identified, and stored in histological cassettes in 70% ethanol.

The analytical steps are summarized below; full methodological details have been reported previously [8].

### 2.5. Micro-Computed Tomography (Micro-CT) Analysis

Non-decalcified calvaria specimens underwent micro-computed tomography (micro-CT) analysis to obtain standardized three-dimensional (3D) images using a SkyScan 1172 system (SkyScan N.V., Kontich, Belgium), operating at a high spatial resolution of 10 µm. Scans were acquired at a voxel size of 6 µm × 6 µm × 6 µm. The X-ray source was operated at 60 kV and 165 µA to optimize image quality and resolution.

The region of interest (ROI) and the volume of interest (VOI) were defined using DataViewer v. 1.4.3 software (SkyScan N.V., Kontich, Belgium). Within each VOI, trabecular bone tissue was quantified according to a predefined grayscale threshold ranging from 80 to 170 (0–255). The structural parameters evaluated were bone volume fraction (BV/TV; bone volume/total volume, %), trabecular separation (Tb.Sp, mm), connectivity density of the trabecular network within the VOI (Conn.Dn), and volume of remaining graft particles (VRP). All measurements were performed by a calibrated examiner (L.M.P.S.) using CTAn v. 1.13.5.1 software (Bruker, Kontich, Belgium).

### 2.6. Confocal Laser Scanning Microscopy

New bone formation was assessed by fluorescence analysis following previously published protocols [16]. Calcein and alizarin labeling represented mineral deposition occurring around days 14 and 30, respectively, thereby allowing assessment of mineralization at two distinct stages of healing. The images obtained from the specimens were subsequently imported into ImageJ software, version 1.43g (National Institutes of Health, Bethesda, MD, USA) for analysis. The distinction between the calcein signal, representing pre-existing bone, and the alizarin signal, representing newly formed bone, provided information regarding the different stages of bone remodeling. In addition, superimposition of the two fluorochrome layers enabled visualization of calcium deposition at both evaluation periods, allowing the progression from pre-existing to newly formed bone to be assessed [16]. New bone formation was subsequently quantified by estimating the mineral apposition rate (MAR). For this analysis, the daily mineralization rate was estimated by measuring the distance between the outer margins of the calcein and alizarin labeling at five distinct points. Measurements were performed with the straight-line selection tool in ImageJ. The resulting value was divided by 16, the number of days between administration of the two fluorochromes.

### 2.7. Histomorphometric and Histopathological Analysis

Histological sections previously analyzed by confocal laser scanning microscopy were stained with Stevenel’s blue and alizarin red for light microscopy. The histometric analysis was performed by a calibrated examiner (U.C.C.M.) using standardized image acquisition and analysis software (LAS EZ v. 4.1.0, Leica Microsystems, Wetzlar, Germany). One representative section of the CSD area of each specimen was selected, and the total defect area was delineated (Total Area, TA). Within this region, the area of newly formed bone (ANB) and the area of remaining graft particles (ARP) were identified and quantified, with ANB expressed as a percentage of the TA.

The histopathological assessment was performed on selected histological sections at higher magnification (×10 and ×20 objectives) to evaluate the structural characteristics of the newly formed bone, with standardized image acquisition across all specimens.

### 2.8. Statistical Analysis

Statistical analysis was performed using jamovi v. 2.6.44 (The jamovi project, Sydney, Australia). The animal was considered the statistical unit, and a significance level of 5% (*p* < 0.05) was adopted for all comparisons. Data are presented as means and standard deviations (SD), and the normality of the data distribution was verified using the Shapiro–Wilk test. One-way analysis of variance (ANOVA) was used to assess between-group differences for all variables, followed by Tukey’s post hoc test.

## 3. Results

### 3.1. Analysis by Micro-Computed Tomography (Micro-CT)

**Figure 1:**
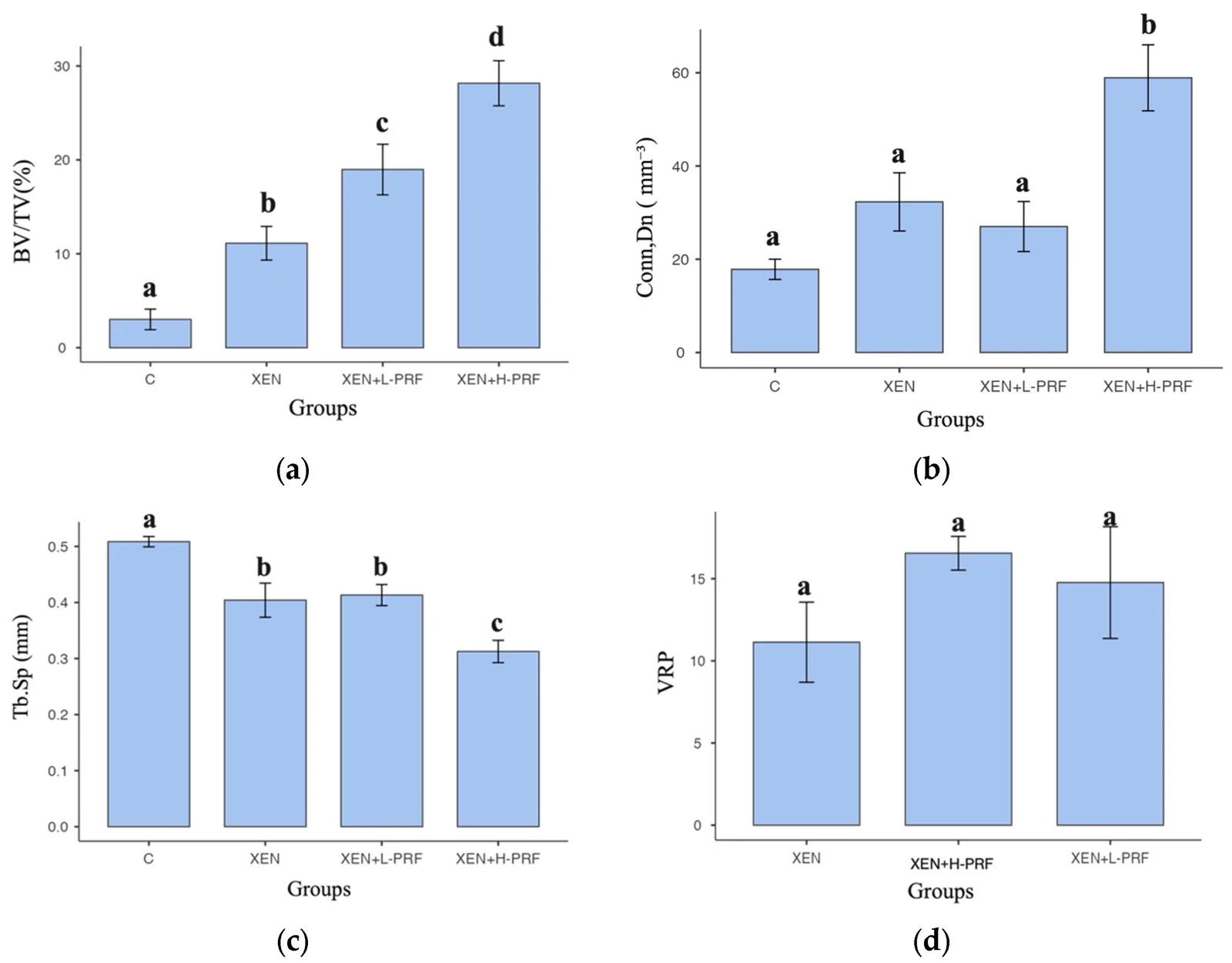
Micro-CT analysis of the rat calvaria. Means and standard deviations (SD) of (**a**) BV/TV, (**b**) Conn.Dn, (**c**) Tb.Sp, and (**d**) VRP for groups C, XEN, XEN+L-PRF, and XEN+H-PRF (*n* = 6 per group). Different letters indicate statistically significant differences between groups (*p* < 0.05).

No animals or data points were excluded from any experimental group or analysis. Figure 1 shows the parameters assessed by micro-CT. All treated groups showed higher BV/TV and lower Tb.Sp values than group C (*p* < 0.05). The XEN+H-PRF group showed significantly higher BV/TV and Conn.Dn values and lower Tb.Sp values than groups C, XEN, and XEN+L-PRF (*p* < 0.05). The XEN+L-PRF group showed significantly higher BV/TV values than group XEN (*p* < 0.05). No significant differences in VRP were found between groups.

**Figure 2:**
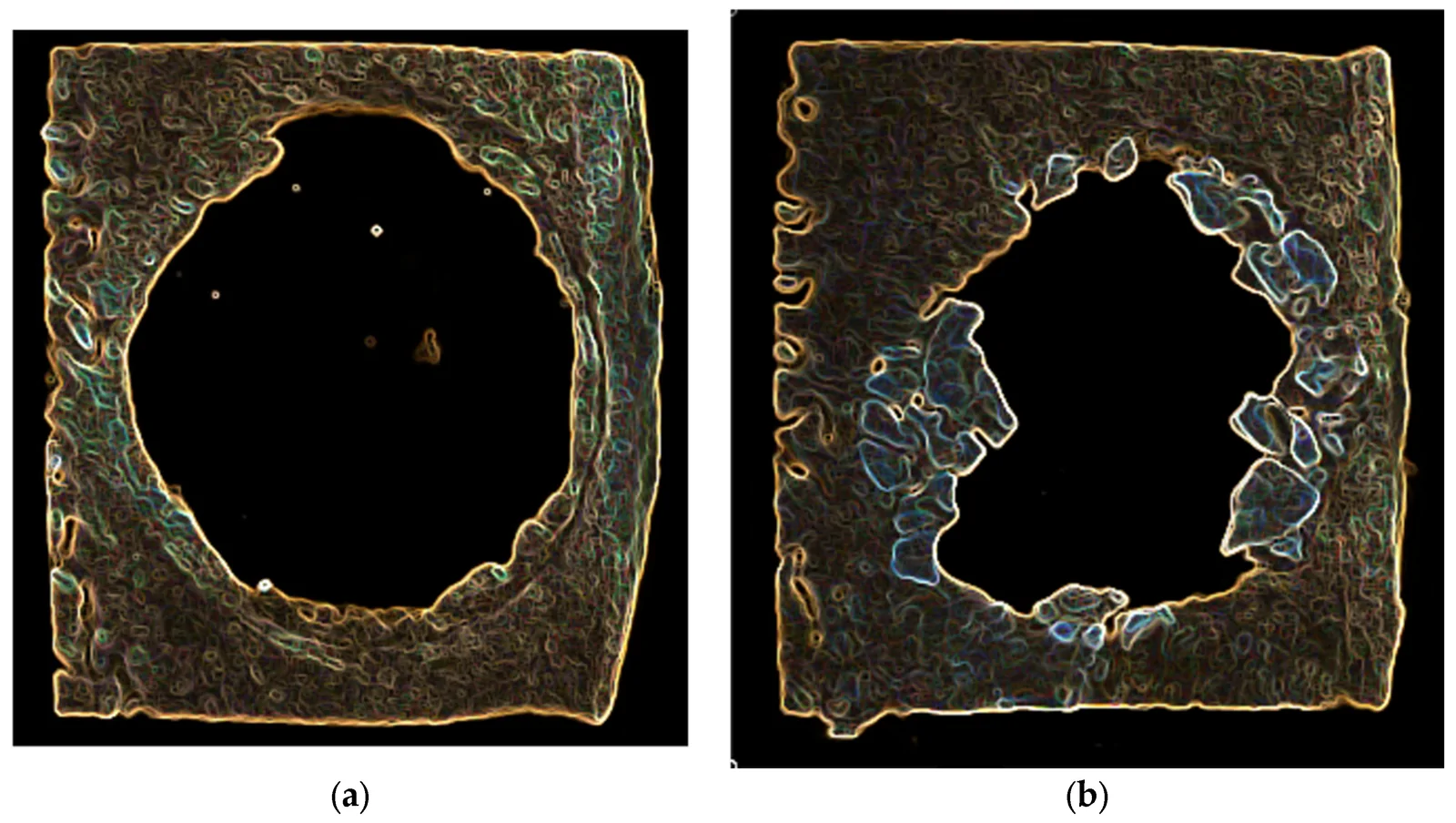

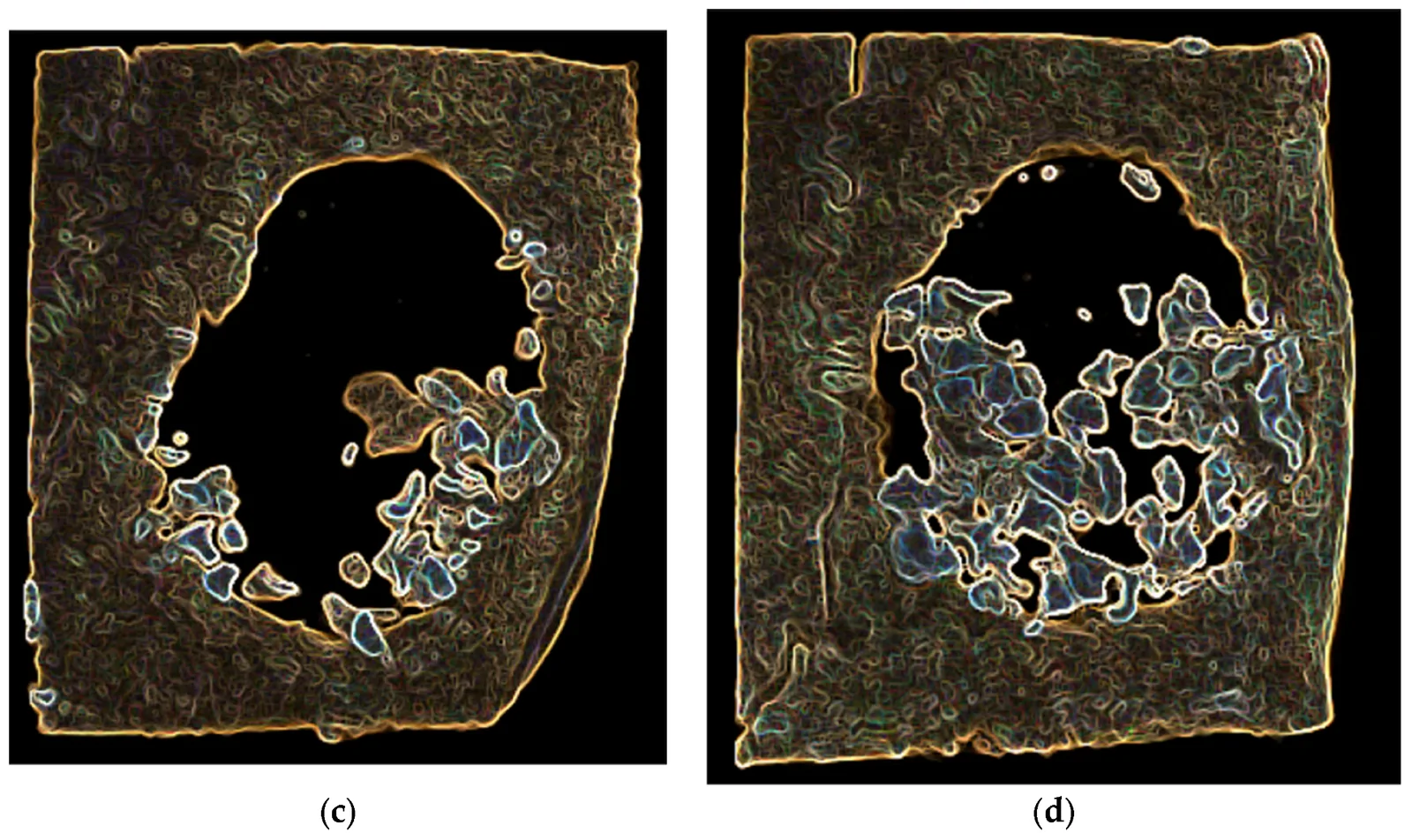
Representative three-dimensional reconstructions of the calvaria in the experimental groups: (**a**) group C; (**b**) group XEN; (**c**) group XEN+L-PRF; (**d**) group XEN+H-PRF.

Representative images of three-dimensional reconstructions obtained from calvaria are illustrated in Figure 2.

### 3.2. Confocal Laser Scanning Microscopy

**Figure 3:**
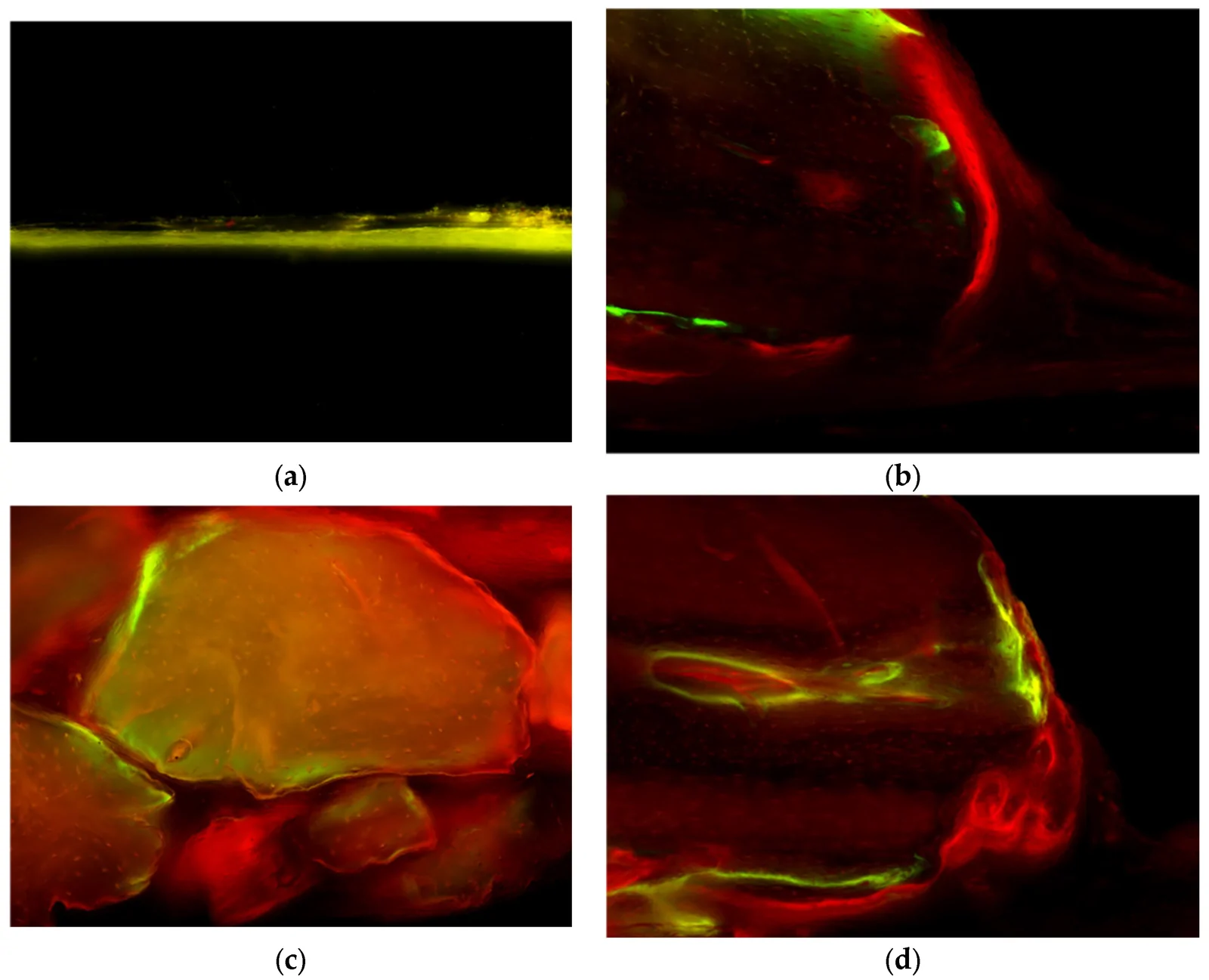

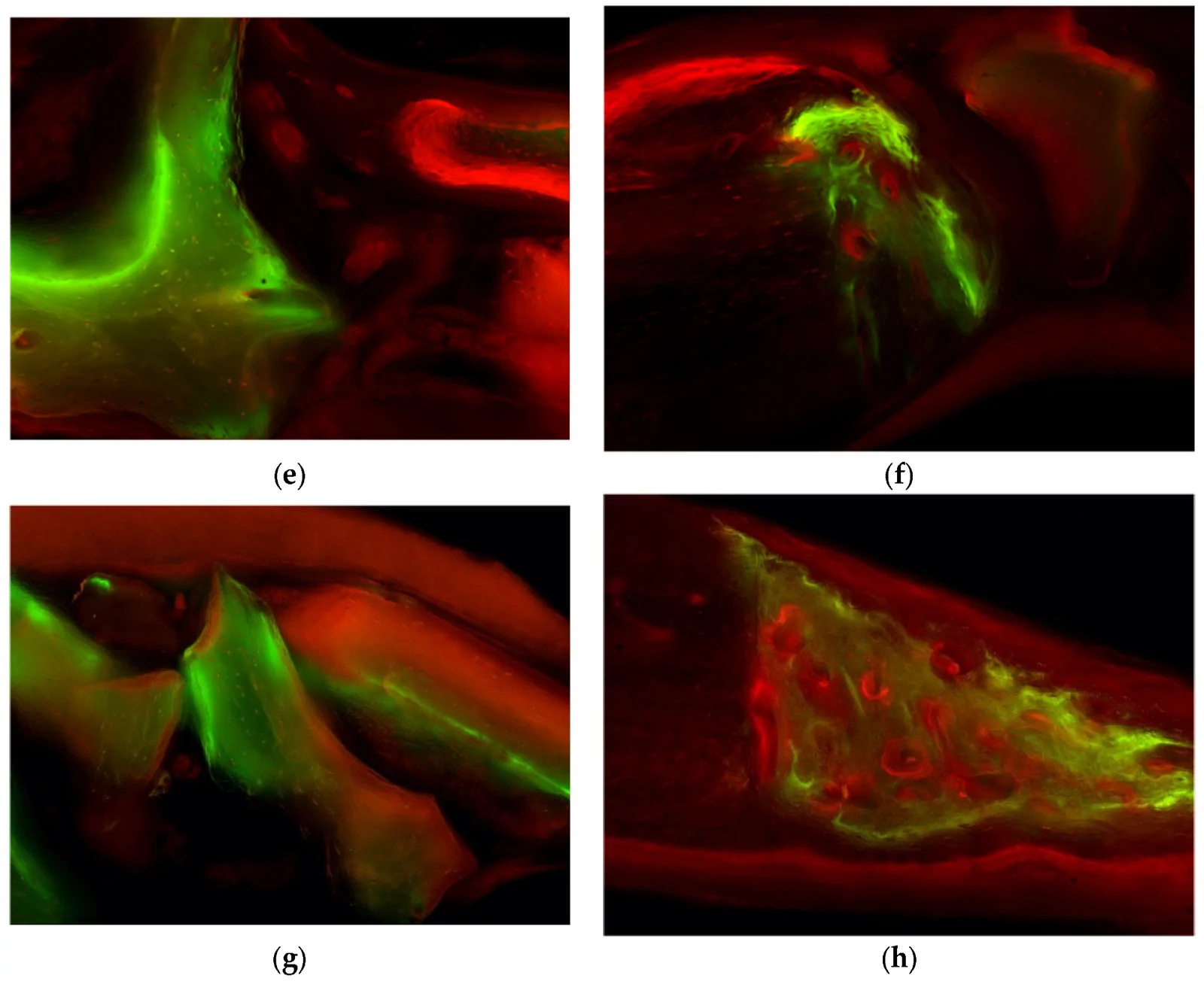
Confocal laser scanning microscopy images showing the superimposition of calcein (green) and alizarin (red) labeling in the experimental groups. (**a**,**c**,**e**,**g**) Representative images of the center of the CSD. (**b**,**d**,**f**,**h**) Representative images of the region near one of the original defect margins. (**a**,**b**) Group C; (**c**,**d**) group XEN; (**e**,**f**) group XEN+L-PRF; (**g**,**h**) group XEN+H-PRF. Original magnification ×10.

Confocal laser scanning microscopy allowed observation of the calcein (injected 14 days after CSD creation) and alizarin (injected 30 days after CSD creation) bands generated by calcium precipitation in the organic matrix. Superimposition of the images allowed pre-existing bone and newly formed bone to be distinguished in the different experimental groups (Figure 3). Group C (Figure 3a,b) and group XEN (Figure 3c,d) showed slow and moderate new bone formation, respectively.

In the XEN+L-PRF group, greater precipitation of calcein and alizarin was observed in the central region (Figure 3e) and near one of the original CSD margins (Figure 3f), as indicated by greater fluorescence intensity.

In the XEN+H-PRF group, the central region (Figure 3g) showed intense calcein and alizarin labeling, indicating a higher rate of new bone formation as well as less resorption of the pre-existing bone. Furthermore, the thickness observed in the central region of the defect was similar to that observed near the margins of the original defect. Near one of the original CSD margins (Figure 3h), more evident calcein and alizarin bands were also observed compared with groups C and XEN.

**Figure 4:**
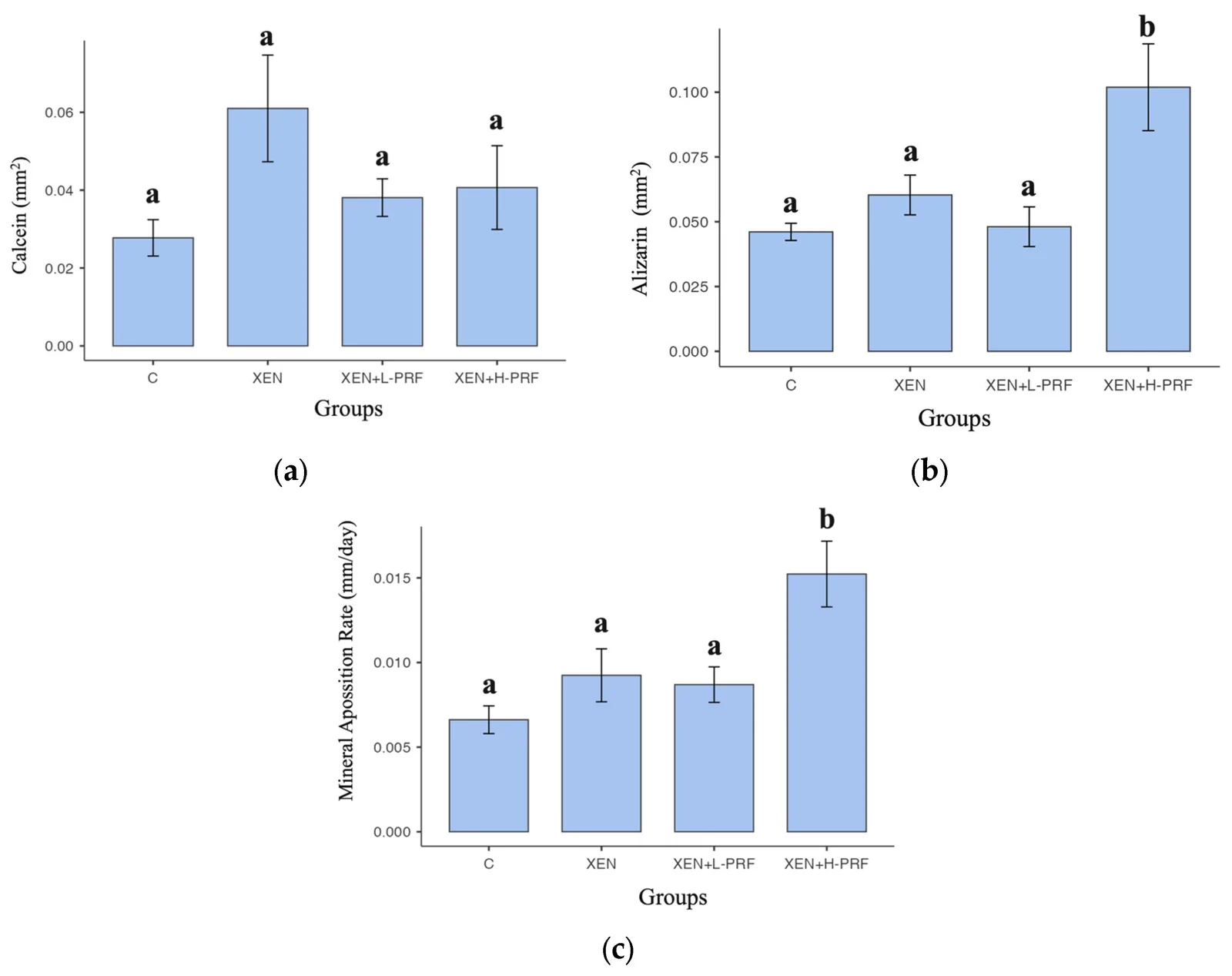
Means and standard deviations (SD) for precipitation of (**a**) calcein and (**b**) alizarin, and for (**c**) the mineral apposition rate (MAR; mm/day) for groups C, XEN, XEN+L-PRF, and XEN+H-PRF (*n* = 6 per group). Different letters indicate statistically significant differences between groups (*p* < 0.05).

Figure 4 presents the results of the confocal laser scanning microscopy analysis. Between-group analysis showed significantly greater alizarin labeling (injected on day 30) in the XEN+H-PRF group than in groups C, XEN, and XEN+L-PRF (*p* < 0.05). The XEN+H-PRF group also showed higher MAR values than the other experimental groups (*p* < 0.05).

### 3.3. Histomorphometric Analysis

**Figure 5:**
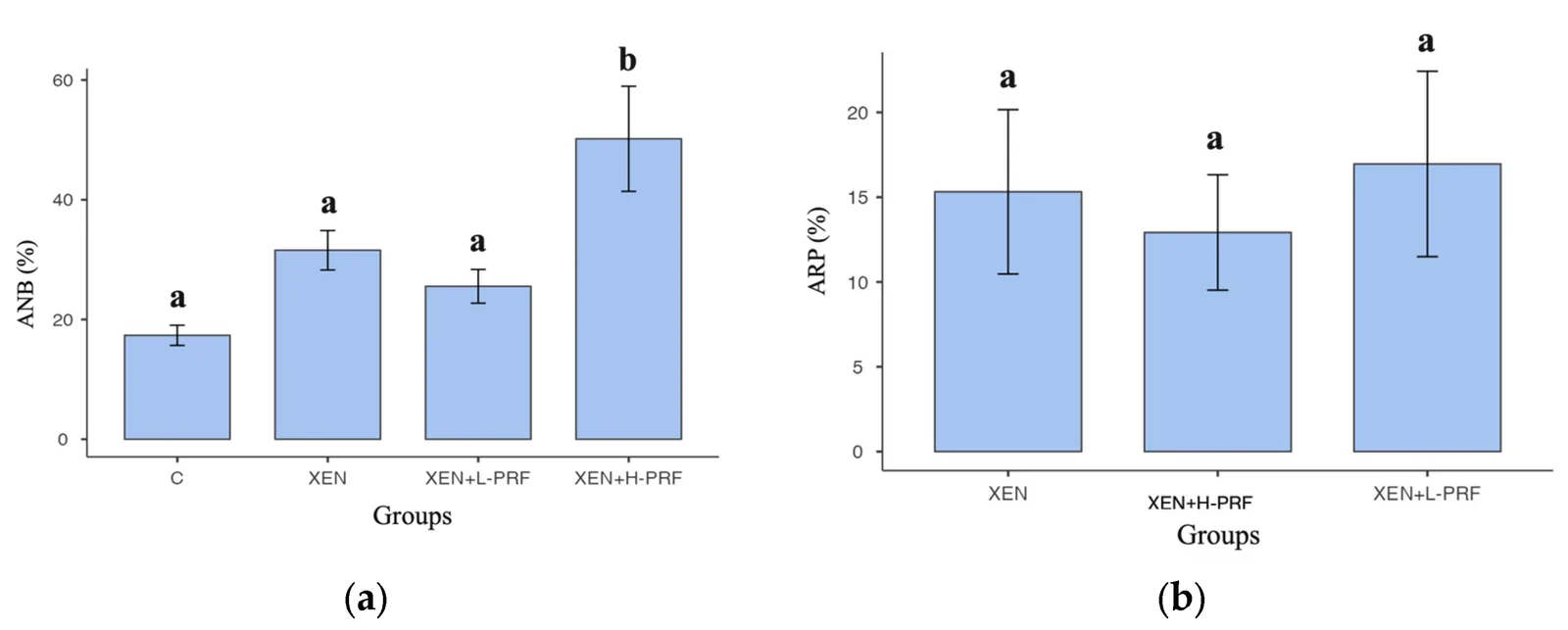
Means and standard deviations (SD) for (**a**) ANB and (**b**) ARP for groups C, XEN, XEN+L-PRF, and XEN+H-PRF (*n* = 6 per group). Different letters indicate statistically significant differences between groups (*p* < 0.05).

The XEN+H-PRF group showed significantly higher ANB values than groups C, XEN, and XEN+L-PRF. No significant differences in ARP were found between groups (Figure 5).

### 3.4. Histopathological Analysis

**Figure 6:**
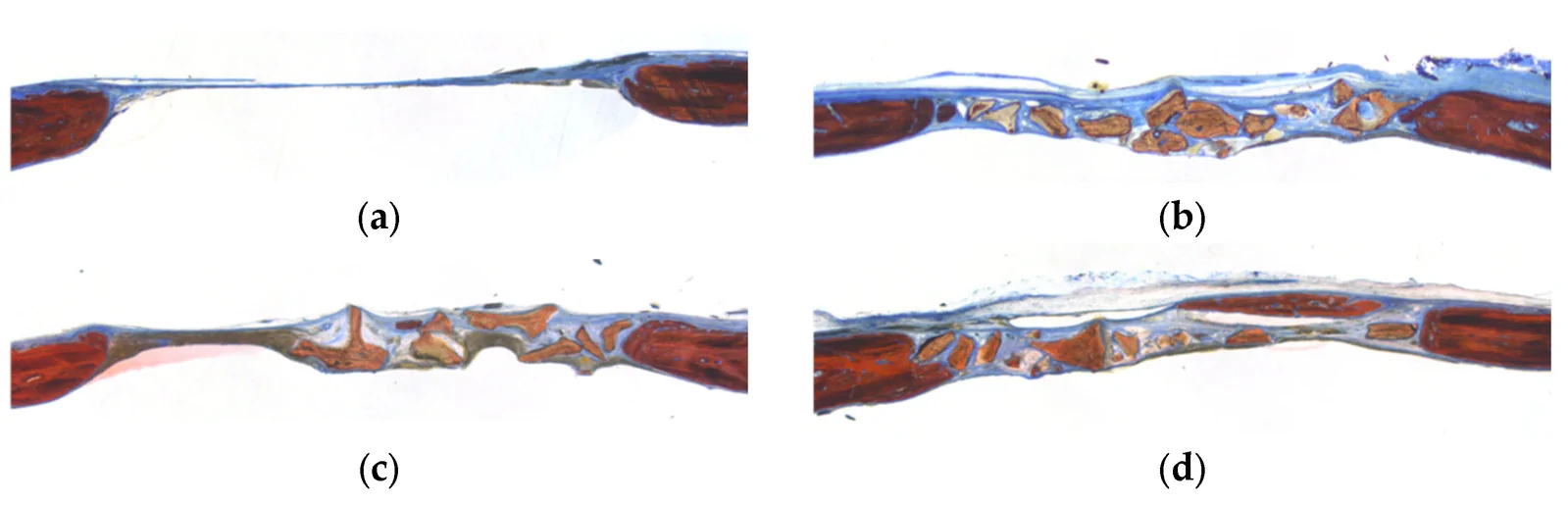
Panoramic images of the histological sections: (**a**) group C; (**b**) group XEN; (**c**) group XEN+L-PRF; (**d**) group XEN+H-PRF. Original magnification ×1.6.

**Figure 7:**
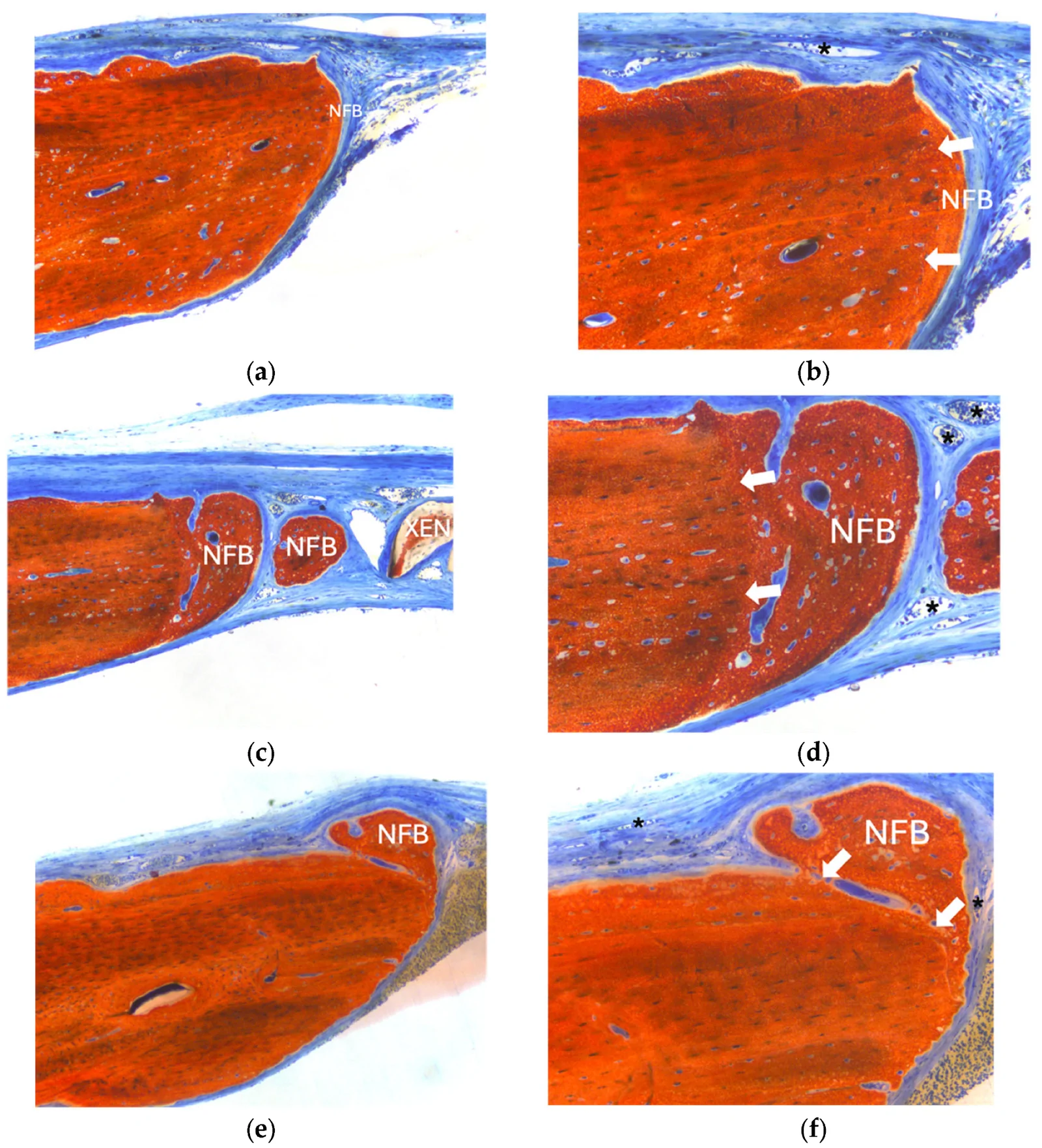

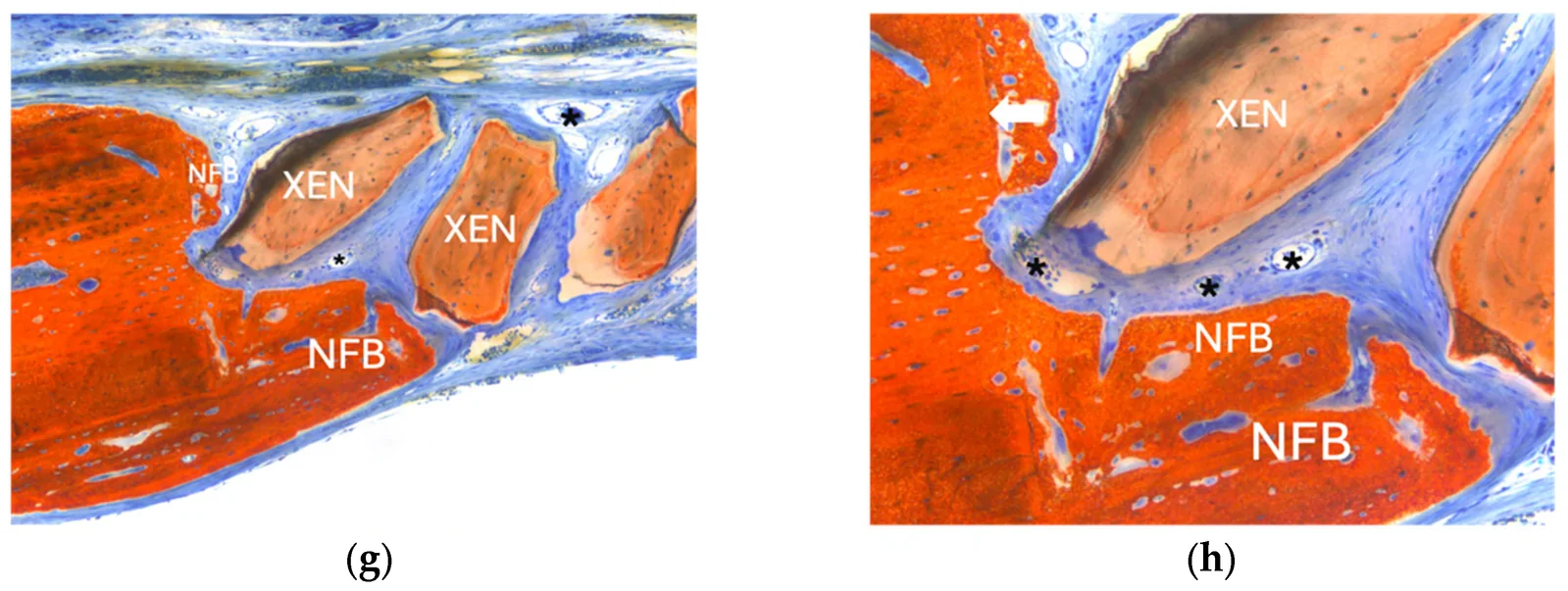
Representative images of the histological sections: (**a**,**b**) group C; (**c**,**d**) group XEN; (**e**,**f**) group XEN+L-PRF; (**g**,**h**) group XEN+H-PRF. Images (**g**,**h**) show regions distant from the margins of the surgical defect. NFB, newly formed bone; XEN, remaining xenograft particles; arrows, margins of the original CSD; asterisks, blood vessels; blue staining (Stevenel’s blue), connective tissue; red staining (alizarin red), bone tissue. Original magnification ×10 (**a**,**c**,**e**,**g**) and ×20 (**b**,**d**,**f**,**h**).

In the control group, the surgical defect was mainly filled with thin connective tissue composed of parallel collagen fibers, and thinner than the original bone tissue of the cranial vault (Figure 6a). Limited new bone formation, together with osteoblasts, blood vessels, and a moderate number of fibroblasts, was observed along the margins of the defect (Figure 7a,b). Bone regeneration was incomplete, suggesting that a longer healing period may be required.

In all specimens of the XEN group, the biomaterial particles and the surrounding connective tissue formed a band of thickness similar to that of the original bone tissue at the defect margins (Figure 6b). The remaining biomaterial particles were surrounded by dense, organized connective tissue, containing osteoid matrix, osteoblasts, and many blood vessels, with some areas of active osteoclast activity. A moderate inflammatory infiltrate, predominantly lymphocytic, was observed, while newly formed and mature bone tissue was observed only at the margins of the defect (Figure 7c,d).

In the XEN+L-PRF group, newly formed bone tissue exhibited few osteoblasts at its margins, indicating an ongoing healing process, while connective tissue without bone differentiation showed numerous thick, well-organized collagen bundles compared with the specimens from group C (Figure 6c). Greater new bone formation was observed at the defect margins, as well as islands of new bone formation within the defect (Figure 7e,f) and marked vascularization, suggesting that the use of XEN+L-PRF promoted improved bone regeneration compared with group C.

In the XEN+H-PRF group, the newly formed bone tissue showed a high density of osteoblasts at its margins, indicating rapid and efficient bone formation (Figure 6d). The connective tissue also showed thicker, well-organized collagen fiber bundles compared with group C (Figure 7g). Most specimens from the XEN+H-PRF group showed greater bone formation along the defect margins than groups C, XEN, and XEN+L-PRF (Figure 7h), with intense new bone formation and islands of bone tissue within the defect. A considerable number of blood vessels were observed throughout the defect area in most specimens from the XEN+H-PRF group.

## 4. Discussion

The present study evaluated the regenerative outcomes associated with two autologous platelet concentrate preparation protocols when combined with a xenogeneic bone graft in critical-size rat calvarial defects. Both PRF-treated groups showed greater bone regeneration than the xenograft-alone group, whereas the XEN+H-PRF group consistently showed the most favorable microtomographic, histomorphometric, and histological outcomes, together with more favorable spatiotemporal bone mineralization kinetics, compared with XEN+L-PRF. Under the experimental conditions evaluated, these findings indicate that the H-PRF preparation protocol was associated with a greater regenerative response when combined with the xenogeneic bone graft.

Autologous platelet concentrates are increasingly used as biological adjuncts because they provide a physiological source of growth factors and immune cells that may support early events involved in bone regeneration [17,18,19,20,21,22]. However, their limited mechanical stability and relatively rapid degradation support their use in combination with slowly resorbing biomaterials, such as xenogeneic bone grafts [10,11,12]. In the present study, both PRF-treated groups showed greater bone regeneration than the xenograft-alone group, consistent with previous evidence indicating favorable outcomes when PRF is combined with bone graft materials [10,11,12,13,18,19,20,21,22,23,24]. The xenogeneic graft provides a mineralized scaffold, whereas the autologous platelet concentrate may contribute to the early healing environment [9,25,26]. The interaction between PRF and the biomaterial may be particularly relevant to the regenerative response, as the biological and mechanical properties of the resulting composite can be influenced by the PRF preparation and incorporation process [27].

In the present study, the XEN+H-PRF group showed the most favorable outcomes compared with the XEN+L-PRF group. The superior regenerative response observed with H-PRF may be related to biological characteristics associated with its preparation protocol. Previous studies suggest that horizontal centrifugation may promote a more homogeneous distribution of platelets and leukocytes within the fibrin matrix, potentially preserving cell viability and influencing the release of bioactive molecules [4,6,8,28,29,30,31]. Differences in fibrin organization and cellular distribution between horizontal and fixed-angle centrifugation may influence the biological behavior of the resulting matrix [4]. These characteristics have been proposed to support angiogenesis, inflammatory modulation [32], osteogenic cell recruitment and differentiation, and antimicrobial activity during the early stages of healing [29,30,33,34,35,36,37]. Consistent with these proposed effects, enhanced bone regeneration has also been observed with H-PRF alone in critical-size calvarial defects [8], whereas previous experimental [25] and clinical [9] studies have reported favorable outcomes when H-PRF is combined with xenogeneic bone grafts. In the present study, the XEN+H-PRF group exhibited the most favorable outcomes for bone volume, connectivity density, mineral apposition rate, and newly formed bone, with blood vessels also observed throughout the defect area. These findings are consistent with the favorable regenerative effects previously proposed for H-PRF [6,8,9,25]. However, the present study did not directly assess the cellular and molecular mechanisms underlying these findings or characterize the PRF samples in terms of fibrin architecture, cellular composition, or growth factor content. Thus, although these characteristics have been described in previous studies [4,28,38], their contribution to the regenerative response observed in the present study could not be directly assessed.

The microtomographic findings further support the superior regenerative potential of H-PRF. Compared with the other experimental groups, XEN+H-PRF exhibited higher bone volume, greater trabecular connectivity, and lower trabecular separation, indicating the formation of a larger and better-connected trabecular network. These findings suggest that H-PRF not only accelerates bone formation but also improves the structural quality of the regenerated tissue. Similar microtomographic outcomes have been reported in both experimental [25] and clinical [9] studies evaluating H-PRF associated with xenogeneic bone grafts for maxillary sinus augmentation. Since xenogeneic bone grafts primarily provide an osteoconductive scaffold [39,40,41,42], the present findings support the concept that H-PRF enhances graft integration by creating a more favorable biological environment for new bone deposition, ultimately resulting in a more mature and functionally organized bone architecture.

Fluorochrome analysis corroborated the microtomographic findings by showing greater mineralized tissue deposition in the XEN+H-PRF group. The greater alizarin labeling and higher mineral apposition rate indicate increased mineral deposition during the later stages of healing, suggesting a more favorable pattern of bone formation in this group. These findings are consistent with a previous experimental study reporting greater alizarin incorporation and enhanced bone formation in defects treated with H-PRF compared with conventional PRF protocols [8]. The combined analysis of calcein and alizarin labeling further demonstrated mineralized tissue deposition at different stages of the healing period, supporting the favorable bone regenerative response observed with H-PRF [8,16]. Although these findings are consistent with enhanced bone formation, the underlying cellular and molecular mechanisms were not directly assessed in the present study.

Histomorphometric and histological findings further corroborated the microtomographic and fluorochrome analyses. The XEN+H-PRF group exhibited a greater amount of newly formed bone and a trend toward fewer residual graft particles, suggesting a favorable pattern of graft integration and replacement by newly formed bone. These findings are consistent with clinical evidence demonstrating increased bone formation and reduced residual biomaterial when H-PRF is combined with xenogeneic bone grafts [9]. Histologically, the XEN+H-PRF specimens exhibited more extensive newly formed bone, with osteoblasts lining the margins of the regenerated tissue and blood vessels distributed throughout the defect area. These findings were consistent with the greater amount of newly formed bone observed by histomorphometric analysis and further support the favorable regenerative response associated with the H-PRF protocol. The observed vascularization and osteoblast distribution are also consistent with the biological effects previously proposed for H-PRF [43]. However, the expression of angiogenic and osteogenic markers, such as vascular endothelial growth factor (VEGF) and runt-related transcription factor 2 (RUNX2), respectively, was not assessed. Further studies evaluating these and other molecular markers are warranted to better characterize the biological responses underlying the observed regenerative effects.

To the best of our knowledge, this is the first preclinical study to directly compare two PRF preparation protocols, H-PRF and L-PRF, when combined with a xenogeneic bone graft. Overall, the present findings highlight the potential importance of PRF preparation parameters in determining its regenerative performance. Although both L-PRF and H-PRF enhanced bone regeneration when combined with a xenogeneic bone graft, H-PRF consistently showed superior structural and histomorphometric bone regeneration outcomes and more favorable spatiotemporal mineralization kinetics.

Some limitations of the present study should be acknowledged. First, only a single healing period was evaluated. Second, H-PRF and L-PRF preparation protocols differed not only in rotor geometry but also in the centrifugation system and processing time. Therefore, despite the similar relative centrifugal force (approximately 700× *g*), the observed differences cannot be attributed exclusively to horizontal versus fixed-angle centrifugation, nor should the findings be generalized to other PRF preparation protocols. Third, cellular composition and viability, growth factor release, angiogenic and inflammatory mediators, and osteogenic molecular markers were not directly assessed. Accordingly, the biological mechanisms proposed to explain the observed histological and morphometric findings should be regarded as hypotheses supported by previous literature rather than mechanisms demonstrated in the present study. Finally, although the rat critical-size calvarial defect is a well-established preclinical model, its biomechanics and healing dynamics differ from those of human bone defects. Thus, the present findings should be interpreted as preclinical evidence and extrapolated cautiously to human guided bone regeneration. Future studies should incorporate molecular and cellular analyses, additional healing periods, and clinically relevant bone defect models to further investigate the regenerative effects of different PRF preparation protocols combined with xenogeneic bone grafts.

## 5. Conclusions

Within the limitations of this preclinical study, the addition of either L-PRF or H-PRF to a xenogeneic bone graft resulted in greater bone regeneration than the xenogeneic graft alone in critical-size rat calvarial defects. Among the treatment groups, XEN+H-PRF demonstrated the most favorable overall performance, exhibiting superior microtomographic structural architecture and histomorphometric outcomes, together with more favorable spatiotemporal bone mineralization kinetics. These findings indicate that the H-PRF protocol evaluated in the present study may represent a promising adjunct to xenogeneic bone grafting for enhancing bone regeneration under the experimental conditions investigated. Further studies are warranted to determine whether these favorable effects are reproducible across different experimental and clinical settings and to establish their relevance to human bone regenerative procedures.

## Data Availability

The original contributions presented in the study are included in the article, further inquiries can be directed to the corresponding author.

## Abbreviations

The following abbreviations are used in this manuscript:

3D	Three-dimensional
ANB	Area of newly formed bone
ANOVA	Analysis of variance
A-PRF	Advanced platelet-rich fibrin
ARP	Area of remaining graft particles
BV/TV	Bone volume fraction (bone volume/total volume)
CEUA	Committee on Ethics in the Use of Animals
Conn.Dn	Connectivity density
CSD	Critical-size defect
H-PRF	Horizontal-platelet-rich fibrin
L-PRF	Leukocyte–platelet-rich fibrin
MAR	Mineral apposition rate
micro-CT	Micro-computed tomography
NFB	Newly formed bone
PRF	Platelet-rich fibrin
RCF_max_	Maximum relative centrifugal force
ROI	Region of interest
RUNX2	Runt-related transcription factor 2
SD	Standard deviation
TA	Total area
Tb.Sp	Trabecular separation
VEGF	Vascular endothelial growth factor
VOI	Volume of interest
VRP	Volume of remaining graft particles
XEN	Xenogeneic bone graft
XEN+H-PRF	Xenograft combined with H-PRF
XEN+L-PRF	Xenograft combined with L-PRF
